# The concept of love in the patchwork family and it’s use in psychotherapy

**DOI:** 10.1192/j.eurpsy.2023.2179

**Published:** 2023-07-19

**Authors:** R. Miętkiewicz, M. Kałaczyńska-Miętkiewicz

**Affiliations:** 1 Polski Instytut Daseinanalizy, Gdynia, Poland

## Abstract

**Introduction:**

The authors - both active psychotherapists and at the same time happily married couple - present bravely a unique case study : theirs own patchwork family. The dwelve deeply into family systems dynamic, analyze the relationship between grandparents, parents and children. The standard roles of father and mother are taken into examination, especially after the youngest baby is born. With wit and humor, the authors define the new working paradigm of patchowork love.

**Objectives:**

Define the new concept of love in patchwork family and redefine the traditional roles.

**Methods:**

Case study.

**Results:**

The result - the new concept of a fully functioning patchwork family with redefined love concept and new roles - enables better understanding and richer life.

**Conclusions:**

We should always seek for love in the families, which come to psychotherapy / psychiatry office for help. Understanding the dynamic of love in the patchwork family is crucial to providing high quality help.

**Disclosure of Interest:**

None Declared

